# Auditory Steady-State Responses for Detecting Mild Hearing Loss in Babies, Infants, and Children: Literature Review

**DOI:** 10.3390/life15071105

**Published:** 2025-07-15

**Authors:** Mariana Ferreira Pires Martins, Caroline Donadon, Piotr Henryk Skarzynski, Ana Júlia Tashiro de Souza, Adriana Neves de Andrade, Daniela Gil, Milaine Dominici Sanfins

**Affiliations:** 1 Post-Graduate Program in Clinical Audiology, Universidade Federal de São Paulo, São Paulo 04044-020, Brazil; 2 Intependent Researcher, Campinas, Brazil; 3 Department of Teleaudiology and Screening, World Hearing Center, Institute of Physiology and Pathology of Hearing, 05-830 Kajetany, Polandmsanfins@unifesp.br (M.D.S.); 4 ENT Department, Maria Curie-Skłodowska University, 20-031 Lublin, Poland; 5 Center of Hearing and Speech Medincus, 05-830 Kajetany, Poland; 6 Department of Otolaryngology, Institute of Sensory Organs, 05-830 Warsaw, Poland; 7 Heart Failure and Cardiac Rehabilitation Department, Medical University of Warsaw, 02-091 Warsaw, Poland; 8 World Hearing Center, 05-830 Kajetany, Poland; 9 Universidade Federal de São Paulo–Escola Paulista de Medicina (UNIFESP), São Paulo 04044-020, Brazil; 10 Department of Speech-Language-Hearing Pathology, Universidade Federal de São Paulo–Escola Paulista de Medicina (UNIFESP), São Paulo 04044-020, Brazil; 11 Department of Audiology, Postgraduate Program in Audiology, Albert Einstein Instituto Israelita de Ensino e Pesquisa, São Paulo 05652-000, Brazil

**Keywords:** auditory evoked potential, children, hearing, electrophysiology

## Abstract

The assessment of hearing in children is important, as hearing deficits can impair child development. The Auditory Steady-State Response (ASSR) is an electrophysiological technique that is able to simultaneously evaluate both ears at four frequencies, making it advantageous for testing children where the test time needs to be as short as possible. The objective of this work was to perform a literature review on the effectiveness of ASSR to gauge hearing thresholds in babies, infants, and children, examining its ability to distinguish mild hearing loss from normal cases. This review used PubMed, Web of Science, and Scopus databases from 2014 to 2024. A total of 1226 articles were identified, although only 16 met the previously established inclusion criteria. It was found that ASSR is a reliable diagnostic tool for babies, infants, and children. Recent work appears better able to distinguish mild hearing loss from normal hearing. One unresolved aspect that needs additional attention is the effectiveness of using bone-conducted stimuli.

## 1. Introduction

Hearing loss that occurs while a child is developing can have a profound effect on the brain. The loss not only disrupts the formation of new synapses and the maturation of central auditory pathways but also accelerates unwanted synaptic pruning [1,2,3]. These neural alterations directly compromise central auditory function, which in turn can significantly impair the child’s communication ability and their quality of social interaction [4]. The integrity and effective functioning of the peripheral and central auditory systems are essential for speech development. Early diagnosis of any impairment is important for ongoing management [5,6].

An auditory diagnosis is usually made first through neonatal hearing screening conducted before discharge in a hospital’s maternity ward. However, in certain cases where the baby, for some reason, is not evaluated at the hospital, auditory function needs to be investigated in an outpatient setting. If poor responses in one or both ears are found, further diagnostic work is then needed [7,8].

To evaluate the auditory function of small babies and infants, electrophysiological methods are called for, since such instruments do not require the active collaboration of the child [9,10]. Currently, the Specific Frequency Auditory Brainstem Response (SF-ABR) and the Auditory Steady-State Response (ASSR) both allow for auditory thresholds to be objectively determined [9].

In SF-ABR, an evaluator identifies the presence of wave V to estimate the auditory threshold. However, the detection of a response is based on identifying peaks and troughs in the waveform and, therefore, introduces an element of subjectivity in interpretation. Testing is usually conducted at four separate frequencies (500, 1000, 2000, and 4000 Hz), with each ear being investigated individually, factors that increase the test time required. The stimuli used can be tone bursts or chirps [11,12,13,14].

By contrast, ASSR investigates auditory thresholds based on statistical tests that allow four frequencies (500, 1000, 2000, and 4000 Hz) to be analyzed simultaneously in both ears [15,16]. Again, tone bursts or chirps are used. This approach reduces the time taken to conduct an exam, and this is especially beneficial for testing children, who find difficulty remaining still for long periods. In addition, ASSR eliminates possible subjective bias in interpreting results [17,18,19].

Early diagnosis of hearing loss is essential for a child’s ongoing development. ASSR allows for a swift and accurate diagnosis, allowing interventions to be initiated as early as possible, ideally before 6 months of age, to maximize treatment outcomes. However, there are still some controversies regarding the use of ASSRs in cases where individuals have hearing close to normal or have only mild hearing loss [17].

With technological advances, hearing assessment equipment now offers greater precision, especially in cases of mild hearing loss [20]. At the same time, given the importance of early detection in infants, it is essential that all the methods used are effective and appropriate [21]. In this context, the present study aims to conduct a systematic review of the literature surrounding the use of the Auditory Steady-State Response (ASSR) in babies, infants, and children who have normal hearing or mild hearing loss. Our goal is to assess the clinical applicability and limitations of the method in early diagnosis in the pediatric population. 

## 2. Materials and Methods

### 2.1. Ethics Considerations

In accordance with Resolution No. 510/2016 of the Brazilian National Health Council, this study was exempt from approval by a Research Ethics Committee because it was based exclusively on a review of previously published scientific material.

### 2.2. Study Design

This systematic literature review aimed to evaluate the applicability of the Auditory Steady-State Response (ASSR) in detecting mild hearing loss or normal auditory thresholds in the pediatric population (children aged 0–12 years). The review followed PRISMA (Preferred Reporting Items for Systematic Reviews and Meta-Analyses) guidelines.

### 2.3. Search Strategy

A comprehensive search was conducted between August and September 2024 across the PubMed, Web of Science, and Scopus databases. Search terms were derived from Medical Subject Headings (MeSH) and included combinations of the following descriptors: auditory steady-state response, objective audiometry, hearing normative, infant, neonatal, diagnostic, auditory evoked potential, and frequency-specific hearing threshold. The Boolean operators “AND” and “OR” were used to combine terms. Table 1 sets out the search strategy.

### 2.4. Eligibility Criteria

Studies were included if they met the following inclusion criteria:

(i) Original research articles;
(ii) Participants aged 0–12 years (covering newborns, infants, and children);
(iii) Evaluation of steady-state auditory evoked potential (ASSR) via air conduction;
(iv) Published in English between 2014 and 2024;
(v) Peer-reviewed publication.

Additionally, the following exclusion criteria were applied:

(i) Studies involving only adults;
(ii) Animal studies;
(iii) Gray literature (dissertations, theses, conference abstracts, or non-peer-reviewed publications;
(iv) Studies that evaluated ASSR exclusively via bone conduction.

### 2.5. Study Selection Process

Two independent reviewers screened the titles and abstracts retrieved from the databases. Articles that met the inclusion criteria underwent full-text review. Discrepancies were resolved through discussion and consensus. The reference lists of the included studies were not screened for additional articles.

### 2.6. Data Extraction and Analysis

A structured spreadsheet was used to include data from the selected studies. The information collected included (i) study title and year of publication; (ii) participant characteristics (age, sex, hearing status); (iii) type and configuration of ASSR stimuli (e.g., CE-Chirp, modulation rate, intensity, frequency range); (iv) equipment used; (v) presence of complementary audiological assessments (e.g., ABR, otoacoustic emissions, tympanometry); and (vi) main results and conclusions. The results are shown in Table 2. No meta-analysis was performed due to heterogeneity in study designs, populations, and outcome measures.

## 3. Results

### 3.1. Study Selection

**Figure 1:**
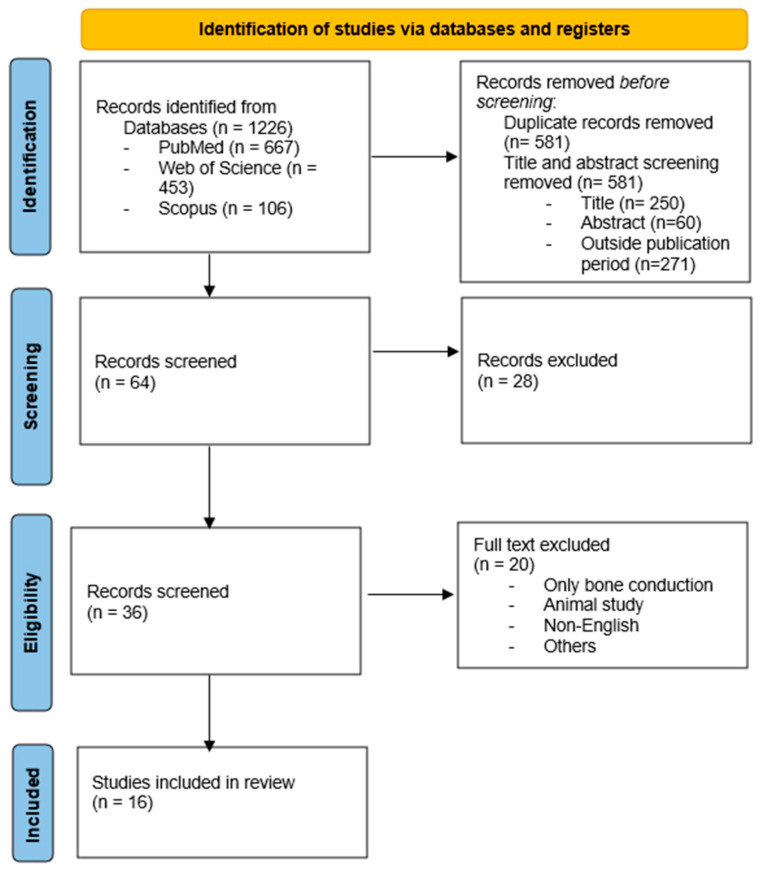
Flowchart for selecting studies.

A total of 1266 articles were retrieved through database searches. After screening titles and abstracts and applying inclusion and exclusion criteria, 16 studies were included in the final review. The process for selecting papers is set out in the PRISMA flowchart (Figure 1).

### 3.2. Sample Characteristics

The studies included samples of babies, infants, and children ranging from 24 h [25] to 11 years of age [30]. Most studies focused on infants younger than 2 years [20,22,23,24,25,26,27,28,29,31,32,33,34,35,36], with a predominance of females [20,23,28,30,33]. Table 2 summarizes the main features of the studies.

### 3.3. Use of Control Groups

Of the 16 studies reviewed, 9 (56%) included a control group in their methodology [23,24,28,29,31,32,34,35,36]. This allowed comparisons to be made between normal hearing individuals and those with hearing impairments [23,28,29,35] and between preterm and full-term infants [24,31,34], as well as to see the effects of age [32,36]. The remaining 6 studies (38%) performed intra-group analyses or focused on just one population group [20,22,25,26,27,33].

### 3.4. Complementary Evaluations

In terms of complementary audiological evaluations, the auditory brainstem response (ABR) was the most frequently used procedure, appearing in 14 (88%) of the studies [20,22,23,25,26,27,28,29,30,31,32,33,34,36]. Evaluation with a click-type stimulus was used in 8 of the studies [20,22,23,25,26,27,32,33], whereas a tone burst was used in just 1 of the studies [34], while a chirp stimulus was also mentioned in 1 other study [29]. In 4 articles (25%), the type of auditory stimulus used in the ABR evaluation was not mentioned [28,30,31,36].

Otoacoustic emissions were used in 10 studies (63%) [20,22,23,24,25,28,30,31,34,35]. Transient evoked otoacoustic emissions were used in 6 of them (38%) [22,23,24,25,28,29,30,35], while distortion product otoacoustic emissions were applied in just 1 [20]. In 2 studies [31,34], use of emissions was mentioned, but the type was not specified.

Immittance testing was reported in 6 studies [20,22,24,26,28,35], most often using a 1000 Hz probe in infants younger than 9 months of age [20,22,24]. In 1 of the studies [20], a combination of 226 and 1000 Hz probes was used. In 3 studies, tympanometry was used, but the probe frequency was not given [26,28,35].

Behavioral hearing assessments were used in 1 study [35], and visual reinforcement audiometry (VRA) was employed in 5 [23,26,27,28,30]. Only 2 study used the cochleopalpebral reflex [34], and another used behavioral audiometry [35].

## 4. ASSR Assessments

### 4.1. Equipment

For ASSR, the Eclipse system by Interacoustics [20,26,27,29,30] was the most commonly reported equipment utilized, followed by the SmartEP from Intelligent Hearing Systems [22,34,35,36] and the Navigator Pro from Biologic-Natus [31,32]. Some 5 studies [23,24,25,28,33] did not specify the device used, presenting a limitation for replication.

### 4.2. Sound Stimuli

As for the characteristics of the stimuli, the CE-Chirp stimulus was the most frequently used (25%) [20,26,27,29], while the traditional tone burst was reported in only 1 study [34]. Regrettably, most of the studies [22,23,24,25,28,30,31,32,33,35,36] failed to report the type of stimulus used (n = 11). Only 1 study clearly stated the use of a second-generation CE-Chirp stimulus [20]. Modulation rates ranged from 67 Hz [23] to 115 Hz [25], with a 90 Hz modulation rate being the most prevalent among the studies.

Although reported stimulus intensity varied from 20 dBnHL [20] to 100 dBnHL [35], 6 studies did not specify the intensity used [23,24,25,27,28,31].

Frequency-specific and ear-specific analyses were conducted in most studies [20,22,26,27,28,29,30,31,34,36]. Usually, studies assessed thresholds at 500, 1000, 2000, and 4000 Hz [20,22,24,25,26,27,28,29,30,31,32,34,36]. All the studies applied air conduction stimulation, in line with the eligibility criteria. However, 25% of the studies also included bone conduction assessments [22,24,25,28], although these were not the primary focus.

### 4.3. Clinical Applicability of ASSR

Regarding the clinical applicability and diagnostic accuracy of ASSR, most of the studies [24,25,26,27,28,30,32,34,35,36,37] supported its use as a reliable method in pediatric populations, especially when estimating auditory thresholds. A smaller portion [23,24,34] recommended its use as a complementary technique alongside other electrophysiological or behavioral methods, while another 3 did not reach definitive conclusions about its effectiveness [29,31,33]. Only 1 study explored the feasibility of using ASSR as a tool for hearing screening [33]. Four studies found high sensitivity and specificity for 500 and 2000 Hz in healthy newborns [33,34,35,36], and some assert that there is commendable sensitivity and specificity; however, Nodarse et al. [33] reported values of 100% for both metrics.

In studies involving preterm infants, some authors reported lower precision and greater variability in ASSR responses compared with full-term peers, particularly at lower frequencies [26,33,36]. However, other studies found no significant differences between groups, suggesting that age-related maturation effects may stabilize responses over time [29,31,34,35]. In children with hearing loss, ASSR was shown to be a consistent method for estimating auditory thresholds and provided results comparable to behavioral tests in many cases [23,26,27,30,35].

## 5. Discussion

In cases of significant hearing loss, the ASSR test is recognized as having a high correlation with behavioral responses [37,38]. However, its applicability in cases where there is a normal hearing threshold, or just mild hearing loss, is still the subject of debate [39]. The present research aimed to focus on ASSR’s abilities in such cases.

The literature review highlighted that one brand (Interacoustics, Middelfart, Dinamarca) was highly represented, justifying its place as a pioneer in the development of the technology. However, the expiry of the original patent has opened up the introduction of alternative models in the market, leading to a proliferation of available systems. Unfortunately, several studies did not mention what sort of equipment they used, and this creates difficulties in trying to replicate them.

In terms of the sound stimuli used, it is pertinent to note that there have been changes and improvements over time. Sound stimuli for assessing ASSR can be classified as first-generation stimuli (stimuli used in the first years after launch of the technology) or second-generation stimuli (the modified stimuli currently available). The CE-Chirp stimulus was introduced as an alternative to the traditional tone burst, and initial studies on its use date back to mid-2014 [40]. When the first studies [41,42] based on the second-generation CE-Chirp appeared, it was discovered that this new sound stimulus allowed responses to be optimized by adjusting the duration and intensity. The low-frequency values, particularly at 500 Hz, exhibited substantial enhancement due to modifications in the second-generation stimulus, resulting in a 20-decibel increase in sensitivity [43]. This improvement was facilitated by adjustments, including the alteration of the standard amplitude modulation stimulus, a phase correction that optimized cochlear delay time, and a frequency shift that permitted the overlap of harmonic responses, thereby significantly enhancing the overall responses [43,44]. Venail et al. [27] observed that second-generation stimuli in ASSR can enhance auditory diagnostics, since the average test time with four frequencies in both ears concurrently is 22.90 min, whereas Sininger [20] reported a test duration of 19.93 min, which is valuable for pediatric diagnosis. The more advanced stimulus model also yielded auditory thresholds that correlated better with Specific Frequency ABR. However, children should be evaluated under natural sleep or sedation to ensure a satisfactory signal-to-noise ratio [44].

Although our literature review covered the years 2014 to 2024, only one study mentioned the use of the second-generation CE-Chirp stimulus [20]. Sininger and colleagues note how this stimulus allows ASSR to identify auditory thresholds at a lower intensity and in a shorter time, with greater consistency with behavioral responses. It is likely that more new studies will be conducted with this stimulus, potentially making ASSR more effective.

Additionally, it is important to consider the auditory stimuli that ASRR values for complex stimuli can be used to estimate how much acoustic speech information is available to the listener. According to Cone and Garinis (2009) [45], electrophoretic measures have the potential to measure children’s perceptual abilities of speech characteristics. Cortical auditory evoked potentials (CAEPs) can be employed in the pediatric population to study the physiological processes and neurological substrates underpinning speech-feature perception in neonates, and they have demonstrated effectiveness in predicting language outcomes [46,47,48].

Another aspect of the sound stimulus is that most researchers have opted to use a 90 Hz modulation rate [20,26,28,30,32,33,34,35,36]. This is appropriate when dealing with children, since such a high rate provides a good correlation with behavioral hearing thresholds—both in children with normal hearing thresholds and in those with some degree of sensorineural hearing loss. However, some researchers warn about limitations of the 90 Hz rate. For example, 40 Hz modulation performs better when hearing at the lower end of the scale (500 and 1000 Hz) is being tested [37,49,50].

This is important because, in general, such frequencies are more difficult to test using other methods, whether behavioral or electrophysiological [51,52]. Furthermore, it is not uncommon for children in a neonatal ICU to have hearing loss at high frequencies and a residual response at lower frequencies, possibly due to the use of ototoxic medications that mainly affect high frequencies [53]. Therefore, an accurate diagnosis at 500 and 1000 Hz is important, since assistance devices need to be set to positively contribute to patient acceptance and rehabilitation [52]. However, if one intends to examine hearing beyond 2000 Hz, using 90 Hz modulation will be more effective [50]. When using high-level intensity stimuli, the use of masking might be considered, since the contralateral ear might be stimulated, discounting the interaural attenuation, which varies from 0 (bone conduction) to 65 dB (air conduction with insert earphones). The use of masking in evoked auditory responses is challenging but needs to be addressed especially in cases of conductive and/or mixed hearing losses [54,55].

Although ASSR responses have been widely investigated in different age groups, there is still disagreement regarding the effect of age on test responses. Thus, Porto et al. [34] and Sousa et al. [24] disagree on whether there are differences between the ASSR responses of premature infants and those of their full-term peers. Porto et al. [34] found no differences between premature and full-term infants, while Sousa et al. [24] reported that the responses of premature infants decreased with time/maturation, and at about 18 months of age, the differences between preterm and at-term babies disappeared. It is important to register that differences between preterm and at-term babies were statistically different but clinically similar. Finally, it is worth mentioning that both studies differed in methodology; the first used only one frequency, and the last used multiple frequency ASSR.

Another work has found no significant differences in the responses of younger babies (5 months) and infants (up to 2 years) [8]. These findings corroborate the work of Van Maanen et al. [36], who found that, from 5 months of age, the responses were broadly the same as those from 2-year-old children. Similarly, when comparisons were made between the responses of babies and adults, it was found that babies had higher thresholds than adults [35]. On the other hand, one study [28] reported that the difference between adults and children is largely restricted to bone conduction stimulation, a finding compatible with those of Alerts et al. [35].

This brings us to the bone conduction issue. One of the most striking aspects of the use of ASSR is different bone conduction responses. According to Casey and Small [28], the assessment of ASSR by bone stimulation is not as accurate as that obtained by means of VRA, since the thresholds obtained by ASSR were, on average, worse. However, there is still much controversy about this. Some authors say that bone conduction stimulation does not seem to be as specific, since they find differences between the values obtained by air conduction and bone conduction. In general, bone conduction values appear to be higher than those obtained by air conduction, but this problem only tends to occur in adults. On the other hand, some researchers have reported that there is no difference between auditory thresholds obtained by air or bone stimulation, either in infants or in adults [22,28]. Most researchers find that, for infants at least, ASSR responses are similar for both stimulation pathways (air and bone) at all frequencies analyzed [20,23,24,25,26,31,34].

Our conclusion is that ASSR assessments via bone are effective in the pediatric population, although in the adult population, they should be used sparingly, since conductive impairments may not be adequately identified. Cases with mild conductive hearing loss may not be identified using ASSR with bone conduction [29].

The differences between air and bone conduction results are important when investigating the type of hearing loss a patient has, especially in the case of babies, infants, and young children [22], whereas conduction stimuli in infants seem to be more intense than the same stimuli in adults, likely due to infant skull maturation [56]. However, most ASSR studies have used air conduction, and so there is a need for new studies exploring whether bone conduction stimulation can contribute to an effective diagnosis.

In their research on ASSR by bone conduction, Small and Stapells [56] found thresholds within normal limits, with an increase for 500 Hz stimuli. Building on this, Hatton et al. [57] proposed a correction factor for 2000 Hz bone conduction ABR in relation to bone conduction ASSR. Further guidance, such as the BSA Early Assessment Guidance [19], offers provisional corrections for calculating dB eHL. These corrections are based on current evidence, accounting for the median difference between ASSR and behavioral thresholds, as well as age-related adjustments.

Similarly, Hulecki and Small [58] observed that behavioral bone conduction minimal response levels using VRA were better in low frequencies compared with high frequencies for infants, like bone conduction ASSRs. This finding suggests the potential for using bone conduction to assess specific frequencies. However, it also highlights the importance of establishing validated reference thresholds to ensure accurate interpretation [29].

To overcome possible deficiencies in the use of bone-conducted ASSR, some researchers have used additional objective assessments such as acoustic immittance measurements [20,22,24,26,28,35]. It is important to mention that, in the pediatric population, particular attention should be given to the probe frequency, since 1000 Hz probes are more effective for younger babies (up to 6 months of age) and 226 Hz probes for older ones. A probe frequency of 1000 Hz is employed for infants due to the mass-dominated admittance of their ears, as a higher-frequency tone is more effective in differentiating diseases from normal middle ear conditions [59,60]. In this review, three research groups used a 1000 Hz probe [20,22,24], but it should be mentioned that broadband tympanometry can also be valuable in assessing whether there are dysfunctions in the tympanic–ossicular system [61,62,63]. Here, the evaluation of pressurized otoacoustic emissions can help in making a differential diagnosis of conductive impairment in children [64] and might be an important diagnostic aid when using ASSR in suspected cases of conductive impairment. However, combined wideband tympanometry and pressurized otoacoustic emissions were not used in the studies we examined.

The main aim of complementary assessments in testing pediatric hearing is to increase diagnostic accuracy and reduce false positives or negatives [65]. They can help differentiate between types and degrees of hearing loss, contributing to a more reliable diagnosis and allowing better monitoring of auditory maturation. A commonly used principle is cross-checking, which refers to the need to use different hearing assessment methods to confirm each finding, reducing the chance of a diagnostic error. This approach ensures that the results obtained by one test are corroborated by another, increasing reliability. In audiology, this is essential, especially for infants and young children, since possible hearing loss needs to be confirmed electrophysiologically and behaviorally.

Cross-checking was observed in all selected articles, where the findings of an ASSR assessment were complemented with other tests. Auditory brainstem response and otoacoustic emissions were the most used tests [20,22,23,25,26,27,28,29,30,31,32,33,34,35,36], probably because they are widely available and can identify hearing deficits in babies, infants, and small children. The assessment of ASSR has been demonstrated by researchers to exhibit a strong correlation with the findings of OAE, as well as with TEOAE [66] and DPOAE [67]. Nevertheless, the responses were more compatible at high frequencies in both cases; thus, the analysis of the responses should be carried out with caution. Porto et al. [34] documented that both ASSR and tone burst ABR at 2000 Hz had good applicability and had the same measurement time (i.e., 20 min).

Furthermore, the integration of methodologies is exceedingly beneficial in detecting patients predisposed to hearing loss, particularly those affected by Congenital Cytomegalovirus (cCMV). In these patients, sensorineural hearing loss is the predominant and debilitating consequence, which may be gradual and late onset; hence, assessment and surveillance are essential. The correlation of ASSR with click and Specific Frequency ABR can yield accurate data, enabling early detection of auditory impairment, hence facilitating the timely administration of suitable drugs to alleviate the advancement of hearing loss [21,68,69].

According to Sininger et al. [20], the ASSR assessment provides lower thresholds and greater diagnostic accuracy than Frequency-Specific ABR. A study by Valeriote et al. [22] investigated how both ASSR and Specific Frequency ABR, applied via air and bone conduction, could detect mild conductive hearing losses. They found that the size of the air–bone gap was insufficient for ASSR to reliably separate normal hearing from mild conductive hearing loss.

François et al. [26] found that the thresholds obtained by ASSR were better by 8–15 dB compared with those obtained through behavioral methods, making it a reliable method for estimating hearing thresholds in children under 6 years of age. However, Panahi et al. [30] observed no differences between the responses obtained by ASSR and those obtained behaviorally. Interestingly, Casey and Small [28] found that ASSR thresholds obtained by bone stimulation were, on average, worse and not as reliable as those obtained through visual reinforcement audiometry (VRA).

In contrast, Panahi et al. [30] found no differences between the responses obtained by ASSR and those obtained by behavioral methods. However, in children with hearing loss, there was no statistically significant difference between the ASSR and VRA thresholds. At the same time, Lu et al. [23] also evaluated this relationship, and found that, in children with normal hearing, mean ASSR thresholds were significantly higher than VRA thresholds, especially at low frequencies.

A pertinent issue to emphasize is the limitation of ASSR in instances of children with auditory neuropathy spectrum disorder (ANSD). Lightfoot et al. [70] indicated that it is not unusual to observe thresholds close to the boundaries of normality in ASSR, even when frequency-specific responses are absent. The occurrence of responses in ASSR may be attributed to sensory artifacts, non-neural responses, and/or short-latency vestibular responses [19]. ASSR evaluation is based on the detection of a peak in a frequency spectrum and receives contributions from multiple generators, which can, unfortunately, result in false-positive results at high levels of sound stimuli in individuals with profound hearing loss due to cochlear nerve malformations, which could be caused by the presence of electrical artifacts [71].

There is still controversy about whether ASSR is a more effective method for determining hearing thresholds than Frequency-Specific ABR. Recommendations for the use of ASSR as being more accurate have already been mentioned [10,20]. Rodrigues et al. [29] underline that ASSR can overcome limitations of Frequency-Specific ABR [72], especially by allowing the evaluation of four frequencies at the same time, making recordings faster. However, there are other researchers who suggest the opposite, favoring the use of Frequency-Specific ABR over ASSR [22,28,29]. Nevertheless, more than half the articles included in this review have concluded that, when applied to the pediatric population, ASSR has better diagnostic efficiency [20,23,24,26,30,31,32,33,35,36]. To reduce the response collection time, a novel assessment technique known as Parallel Auditory Brainstem Response (pABR) has recently been suggested; however, it still needs to be evaluated in the pediatric population due to its low-frequency restrictions [73].

## 6. Conclusions

This research has confirmed the effectiveness of ASSR as a diagnostic tool for babies, infants, and children; however, ABR is still considered the gold standard within the audiological diagnostic process. Technological innovations such as second-generation CE-Chirp stimuli have led to better detection sensitivity, facilitating the differential diagnosis of mild hearing loss or normal auditory threshold. Although there are still large uncertainties associated with the use of bone conduction, ASSR appears to be a very effective tool in the pediatric population we studied. Furthermore, it is relevant to mention that, in cases where children do not sleep, ASSR is not a viable assessment option; therefore, evaluation through cortical auditory evoked potentials could be employed.

## Figures and Tables

**Table 1:** Search strategy used in databases based on MeSH descriptors and Boolean operators.

Search ID	Combination Search Terms (MeSH)	Database		
		PubMed	Web of Science	Scopus
1	(“Auditory Steady State Response” AND “Objective Audiometry”) OR (“Hearing Normative” AND “Infant”)	311	250	22
2	(“Auditory Steady State Response” AND “Objective Audiometry”) OR (“Hearing Normative” AND “Neonatal”)	151	150	5
3	“Auditory Steady State Response” AND “Objective Audiometry” AND “Diagnostic” AND “Infant”	59	6	14
4	“Auditory Steady State Response” AND “Auditory Evoked Potential” AND “Neonatal” AND “Infant”	68	8	43
5	“Auditory Steady State Response” AND “Frequency-specific Hearing Threshold” AND “Neonatal” AND “Infant”	10	8	2
6	“Auditory Steady State Response” AND “Auditory Threshold” AND “Neonatal” AND “Infant”	55	14	11
Total number of articles		654	436	97

**Table 2:** Characteristics and results achieved of studies included in the present review.

Author/Ref.	Participants	ASSR Parameters	Complementary Audiological Tests	Objective	Main Findings	Author’s Conclusions
Valeriote et al. [22]	EG: 64 infants (0–6 mo) CG:—none	Type: Multiple Freq 500–4000 Hz Stim: AC and BC Equip: IHS Mod rate: 78–101 Hz Stimulus:—not provided Intens: 30 dB	TOAE, Tymp (1 kHz), Click-ABR	To compare ASSR (AC/BC) with Click-ABR in detecting conductive HL in infants	BC thresholds were similar between groups. Greater AC–BC gap at 500 Hz in conductive HL. ASSR thresholds showed more variability	ASSR showed greater variability and exaggerated the AC–BC gap. Click-ABR (AC) had better sensitivity/ specificity for detecting conductive HL
Lu et al. [23]	EG: 15 children with HL (1–6 y; 11 F) CG: 10 children with NH (1–8 y)	Type: Multiple Freq 250–4000 Hz Stim: AC Equip:—not provided Mod rate: 67–95 Hz Stimulus:—not provided Intens:—not provided	VRA, TOAE, Click-ABR	To compare thresholds using ASSR, CM, and VRA in children with HL and NH	ASSR thresholds slightly higher than VRA in NH. VRA outperformed ASSR in HL	ASSR thresholds slightly higher than VRA. No significant difference in ASSR/VRA threshold gap between groups.
Sininger et al. [20]	EG: 102 children (7–80 mo; 58 F) CG:—none	Type: Multiple Freq 500–4000 Hz Stim: AC Equip: Eclipse Mod rate: 90 Hz Stimulus: CE-Chirp Intens: 20 dB	Tymp (226 Hz and 1 kHz), DPOAE, Click-ABR	To compare ASSR and Click-ABR thresholds using optimized stimuli and detection algorithms	ASSR thresholds were lower than Click-ABR	ASSR effective for NH detection; faster audiogram predictor
Sousa et al. [24]	EG: 33 preterm CG: 30 full terms	Type: Multiple and Single Freq 500–4000 Hz Stim: AC and BC Equip:—not provided Mod rate: 111.41 Hz (LE), 115 Hz (RE) Stimulus:—not provided Intens:—not provided	Tymp (1 kHz), TOAE	Comparing ASSR thresholds in preterm and term infants at two time points	Higher thresholds initially in preterm. No difference at 18 mo	ASSR responses stabilize by 18 mo; useful for neonatal diagnostics
Torres-Fortuny et al. [25]	EG: 69 newborns (1–16 d) CG:—none	Type: Multiple and Single Freq 500–4000 Hz Stim: AC and BC Equip:—not provided Mod rate: 111.41 Hz (LE), 115 Hz (RE) Stimulus:—not provided Intens:—not provided	TOAE, Click-ABR	To evaluate ASSR amplitude responses using single or simultaneous AC and BC stimulation in newborns	No ASSR amplitude differences between single and simultaneous AC and BC stim	Both stim types yield stable ASSR amplitudes in newborns
François et al. [26]	EG: 175 children (0–6 y) CG: -none	Type: Multiple Freq 500–4000 Hz Stim: AC Equip: Eclipse Mod rate: 80 Hz Stimulus: CE-Chirp Intens: 75 dB	Tymp (ND), VRA, Click-ABR	To assess whether ASSR responses are dependable compared with Click-ABR and behavioral field audiometry	ASSR correlated with behavioral and ABR thresholds. ASSR thresholds were better (8–15 dB difference)	ASSR is as dependable as click-ABR for 2–4 kHz in children <6 y. Valuable when behavioral thresholds are not feasible
Venail et al. [27]	EG: 32 infants (5.2–7.4 mo) CG:—none	Type: Multiple Freq 500–4000 Hz Stim: AC Equip: Eclipse Mod rate:—not provided Stimulus: CE-Chirp Inten:—not provided	VRA, Click-ABR	To access bilateral simultaneous ASSR using NB CE-Chirps in infants	ASSR correlated well with Click-ABR and VRA. No difference between VRA and ASSR mean thresholds	NB CE-Chirps offer reliable rapid threshold estimates, especially at low frequencies
Casey and Small [28]	EG: 23 infants NH (6.5–19 mo; 12 F) CG: 12 adults NH (17–50 y; 10 F)	Type: Multiple Freq 500–4000 Hz Stim: AC and BC Equip:—not provided Mod: 78–101 Hz Stimulus:—not provided Intens:—not provided	Tymp (ND), VRA, TOAE, ABR-SF (ND)	To compare AC/BC thresholds via ASSR and behavioral methods in infants and adults	BC ASSR thresholds were worse than VRA thresholds. Hearing thresholds in infants and adults for AC and BC were similar at all frequencies.	Differences in infant–adult and AC–BC thresholds are greater in the ASSR assessment compared with the behavioral auditory assessments.
Rodrigues et al. [29]	EG: 30 full-term infants NH (6.5–19 mo; 16 M) CG: 10 young adults (23–30 y; 5 F)	Type: Multiple Freq 500–4000 Hz Stim: AC Equip: Eclipse Mod rate:—not provided Stimulus: CE-Chirp Intens: 50 dB	TOAE, ABR-Chirp	To estimate AC thresholds via ASSR to NB CE-Chirp and compare across age groups	Normal thresholds in both groups, except at 500 Hz (infants > adults).	ASSR is useful for estimating thresholds, with age-related effects at 500 Hz.
Panahi [30]	EG: 26 children (2.4–11 y; 13 F) CG:—none	Type: Multiple Freq 500–4000 Hz Stim: AC Equip: Eclipse Mod rate: 90 Hz Stimulus:—not provided Intens: 20 dB	VRA, TOAE, ABR-SF (ND)	To compare ASSR and VRA thresholds in children with a history of neonatal hyperbilirubinemia	The mean difference between VRA and ASSR thresholds was 1.5–8.48 dB in children with HL and 7.29–13.95 dB in children with NH.	90 Hz ASSR provides reliable threshold estimates in this population.
Ribeiro et al. [31]	EG: 21 NH premature infants (<37 weeks; 11 F) CG: 56 NH full-term infants (>38 weeks; 13 F)	Type: Multiple Freq 500–4000 Hz Stim: AC Equip: Biologic Mod rate: 91.406–96.094 Hz (LE); 99.094 Hz (RE) Stimulus:—not provided Intens:—not provided	OAE, ABR-SF (ND)	To estimate hearing thresholds using ASSR in term and premature infants with NH	Higher thresholds and variability at 500 and 4000 Hz in preterm.	ASSR effective in both groups; preterm showed more variability.
Choi et al. [32]	EG: 44 infants (3d) CG: 15 infants (3–15 weeks)	Type: Multiple Freq 500–4000 Hz Stim: AC Equip: Biologic Mod rate: 80.08–94.73 Hz (LE); 78.13–91.80 Hz (RE) Stimulus:—not provided Intens: 50 dB	Click-ABR	To examine ASSR phase stability in newborns using different modulation rates	No phase differences by age. Phase polarization methods can improve detection rates and test speed.	ABR phase responses evoked by exponentially amplitude-modulated tones are stable.
Nodarse et al. [33]	EG: 50 infants (7–18 d; 22 F) CG: —none	Type: Multiple Freq 500 and 2000 Hz Stim: AC Equip:—not provided Mod rate: 81 Hz (LE); 97 Hz (RE) Stimulus:—not provided Intens: 60 dB	Click-ABR	To evaluate a semi-automatic hearing screening test using ASSR	Hearing thresholds reaching 100% detectability for 45 and 50 dB. The screening test achieved 100% sensitivity and 96% specificity.	ASSR is a feasible screening tool in healthy newborns.
Porto et al. [34]	EG: 17 premature infants (<37 weeks; 12 F) GC: 19 full-term infants (>37 weeks; 11 M)	Type: Multiple Freq 500–4000 Hz Stim: AC Equip: IHS Mod rate: 93 Hz (LE); 97 Hz (RE) Stimulus: TB Intens: 80 dB	CPR, OAE-ND, ABR-TB	To compare ABR-TB and ASSR responses in preterm vs. term infants	The responses of preterm and full-term infants were similar. ASSR was slightly longer (test time) in preterm.	Both approaches are applicable in clinical practice. Premature infants require more time for ASSR.
Alaerts et al. [35]	EG: 70 infants at risk for HL (4–19 weeks; 40 M) CG: 16 NH adults (20–29 y; 13 F)	Type: Multiple Freq 500–4000 Hz Stim: AC Equip: IHS Mod rate: 82–106 Hz (LE); 86–110 Hz (RE) Stimulus:—not provided Intens: 20 to 100 dB	Tymp (ND), TOAE, BA	To compare ASSR thresholds between infants and adults with NH	Normal ASSR thresholds were higher in infants compared with adults. Strong correlation between ASSR and BA.	The thresholds of normal infants were higher than those of adults with NH when comparing ASSR. ASSR thresholds by AC showed good correlations in infants with varying degrees of HL.
Van Maanen et al. [36]	EG: 22 babies (<6 mo) CG: 32 babies (>6 mo)	Type: Multiple Freq 500–4000 Hz Stim: AC Equip: IHS Mod rate: 81–105 Hz (LE); 77–100 Hz (RE) Stimulus:—not provided Intens: 70 dB	FS-ABR (ND)	To evaluate use of 80 Hz ASSR for threshold estimation in infants	Consistent thresholds at 49, 45, 36, and 32 dB HL across the frequencies. No differences in results for younger v. older infants.	ASSR effective for confirming NH in both ears at multiple frequencies.

Legend: ABR: auditory brainstem response; AC: air conduction; ASSR: Auditory Steady-State Response; BA: behavioral audiometry; BC: bone conduction; CE: Chirp-Evoked; CG: control group; CPR: cochleopalpebral reflex; d: days; dB: decibel; DPOAE: distortion product otoacoustic emissions; EG: experimental group; Equip: equipment; F: female; Freq: frequency; FS: frequency-specific; HL: hearing loss; Hz: hertz; Intens: intensity; LE: left ear; M: male; mo: months; Mod rate: modulation rate; ND: not detailed; NH: normal hearing; NB: narrowband; OEA: otoacoustic emissions; PDOEA: distortion product otoacoustic emission; RE: right ear; Stim: stimulation; TOAE: transient otoacoustic emissions; Tymp: tympanometry; VRA: visual reinforcement audiometry; y: years.

## Data Availability

No new data were created or analyzed in this study. Data sharing is not applicable to this article.

## Abbreviations

The following abbreviations are used in this manuscript:

ABR	auditory brainstem response
AC	air conduction
ASSR	Auditory Steady-State Response
BA	behavioral audiometry
BC	bone conduction
CE	Chirp-Evoked
CE-Chirp	Chirp-Evoked Stimulus
CG	control group
CPR	cochleopalpebral reflex
d	days
dB	decibel
dB eHL	decibel estimated hearing level
dB HL	decibel hearing level
DPOAE	distortion product otoacoustic emissions
EG	experimental group
Equip	equipment
F	female
Freq	frequency
FS	frequency-specific
FS-ABR (ND)	Frequency-Specific Auditory Brainstem Response (with type of stimulus not detailed)
HL	hearing loss
Hz	hertz
ICU	intensive care unit
Intens	intensity
LE	left Ear
M	male
MeSH	Medical Subject Headings
mo	months
Mod Rate	modulation rate
NB	narrowband
NH	normal hearing
OAE	otoacoustic emissions
OAE-ND	otoacoustic emissions—not detailed
PDOAE	distortion product otoacoustic emission
PRISMA	Preferred Reporting Items for Systematic Reviews and Meta-Analyses
RE	right ear
SF-ABR	Specific Frequency Auditory Brainstem Response
Stim	stimulation
TOAE	transient otoacoustic emissions
Tymp	tympanometry
Tymp ND	tympanometry–type of probe not detailed
VRA	visual reinforcement audiometry
y	years
